# Designing an Energy-Efficient Transportation Network to Transport Perishable Crops: An Aggregated VRP and X-means Clustering Approach

**DOI:** 10.1016/j.heliyon.2023.e19692

**Published:** 2023-08-30

**Authors:** Joydev Karmakar Rahul, Souvik Chakraborty, Nabila Khayer, Md. Foysal Uddin, Maliha Rajwana Haque

**Affiliations:** Department of Industrial & Production Engineering, Rajshahi University of Engineering & Technology, Kazla, Rajshahi-6204, Bangladesh

**Keywords:** Perishable Crops, Aggregated Vehicle Routing Problem, X-means Clustering, Aggregation Points, Cost Optimization

## Abstract

Almost all perishable crops deteriorate due to improper and tardy transportation and storage. Vehicle Routing Problem, or VRP, might be of great aid since it takes into account a number of aspects of any transportation and storage issues and optimizes them in such a way as to reduce the overall cost of the carrier. This study attempts to widen the scope of the commonly used VRP model by including traffic and energy consumption features and transforming it into the Aggregated Vehicle Routing Problem (AVRP). Traditional VRP focuses on minimizing distance. Generally, it is unable to find out the optimal number of aggregation points required to serve a system. So, cost optimization of the AVRP approach was designed with two specialized steps. Firstly, the destination data are divided into multiple clusters employing the X-means clustering. And then the best route was found to execute the delivery thus minimizing cost, required time, and carbon footprint. The study was implemented on the Chattogram zone and discovered that the optimal number of aggregation points (AP) required to serve Chattogram is only three namely- AP 1, AP 2, and AP 3. VRP analysis was stretched further with AVRP model using AP 1 and found to reduce the operating cost by 10.96%.

## 1 Introduction

Being an agricultural nation, one would anticipate that food and vegetable management would be done in a way that minimized losses. However, the estimated annual loss of fruits and vegetables in Bangladesh is 23.6–43.5% [1]. So, almost half of what is produced goes to waste due to improper management and myopic planning of the post-harvest movement. The economic losses that also stem from this are also significant as pointed out by one research [2]. Fruits and vegetables that have rotted after being harvested result in an annual loss of about 3392 crore BDT. The improper management of the system may include inefficient aggregation, distribution, forecasting, bullwhipping, storing, and transportation and these are major contributors to the waste of perishable crops. Due to insufficient knowledge of handling, moving, storing, and packing post-harvest goods properly especially perishable crops, thousands of tons of fruits and vegetables are thrown away each year.

Lack of appropriate and timely transportation and storage causes almost all perishable crops to degrade. Most perishable crops require cold storage, but there aren't enough facilities. If there is a strong transportation network, food loss will be minimal, and there won't be any issues with hunger. Therefore, loss mitigation strategies need to be strengthened. To alleviate this problem, the Vehicle Routing Problem or VRP could be of tremendous assistance since it encompasses a number of factors of any transportation and storage problems and optimizes them to that effect so the total cost of carrier is minimized. Among the various types of VRP, this will address the specific attributes of perishable crops.

Energy efficiency is the key to sustainability. An energy-efficient transportation network is a system that is designed to minimize the use of energy while providing transportation services. Such a system can reduce the overall energy consumption and carbon emissions associated with transportation, which is one of the major sources of greenhouse gas emissions. It can also bring additional advantages including cost reduction, reduced lead time, better mobility etc. Sustainability of any transportation model requires attention to fuel consumption and sometimes the total emission of CO_2_. With the help of VRP, one can easily include fuel consumption and other green transportation to address the ever-growing concern of sustainability. Also, the movement of any perishable products demand the proper maintenance of the cold chain and an overview of the whole supply chain in terms of its shelf life. An appropriate number of aggregation points can reduce the total cost and won't affect the perishable product with shorter shelf life. The implementation of any VRP model can easily be deflected by the traffic characteristics of the chosen city.

An attempt has been taken in this research to broaden the scope of the widely used Vehicle Routing Problem (VRP) by incorporating traffic and energy consumption characteristics and transforming it into the Aggregated Vehicle Routing Problem (AVRP) model. In contrast to the VRP concept, the AVRP may reduce the overall energy usage of refrigerated vehicles. The VRP model focuses on the tank's fuel capacity but ignores the consumption rate. The number of aggregation points employed in the network to maintain cold storage and distribution facilities is a crucial component of the model. In addition, we have highlighted how the X-means clustering technique is used to determine the ideal number of aggregation sites.

## 2 Literature review

The improvement of the performance of any transportation model is examined from various angles and on different factors. One can find a plethora of routes and strategies to optimize their transportation model through various research. But the availability of research in the area of optimizing the transportation model for perishable foods or in a sense the supply chain of perishable foods is scarce. All the research can also be categorized from which point of view the factors are considered i.e., are they considered from the client or grower's perspective or from the customer's perspective? This section tries to explore the inventory management of the Supply Chain for perishable products, how vehicle routing problem (VRP) is used in this context, and also considers the planning of urban freight transportation.

For example, Ahumada and Villalobos [3] examined the stage of operational planning decisions for maximizing revenues from the grower's side. The level of decision-making is implemented during the harvest season and the main factors that were considered include labor costs, labor availability, price dynamics, weather, quality, cost, and a trade-off between cost and quality. It is often common in any transportation model that the optimization is directed toward maximizing revenues and in the case of perishable products a trade-off between cost and quality i.e., freshness.

Rossi et al. considered how intermodal transportation namely rail-road, can be implemented for perishable foods acknowledging the fact that in reality rail transportation is very slow and not very practical despite its advantages in cost and environmental sustainability [4]. Their case study reveals that this new paradigm can also be economically sustainable depending on consumer preferences and markets where the demand for product variety is high.

While considering multimodal transportation, Leleń and Wasiak [5] found how to increase the efficiency of the transportation models for perishable products by accurately selecting transport technology specifically for freight cargo given the transportation task. They developed this model by considering a number of criteria; most of which are related to cargo safety. The selection of technology can be influenced by transported time, maximum and minimum air temperature, cargo dimension, resistance to mechanical damage, etc. which are related to safety (18 factors) and one factor regarding transportation cost.

Shifting to a more unique situation where the interest of both clients and carriers of perishable cargoes are considered; Muzylyov et al. [6] provided a unique perspective. This approach is based on this integrated business methodology with technological features to find a rational supply chain for perishable foods.

It is undeniable that the vulnerability of the supply chain of perishable products stems from the fact that it is time and temperature sensitive [7]. Keeping that in mind Haji et al. [8] attempted to find out how different technological implementation can improve the supply chain of perishable foods and solidifies the fact that it is overall beneficial to implement technology in perishable SC. They considered a number of technologies ranging from widely used such as RFID, Blockchain, and IoT to emerging technology such as 3DP, UAV, and autonomous vehicles.

Taking a closer look at the inventory management of SC of perishable products Chaudhary et al. [9] investigated various articles. Their findings indicate that the most important parameters in these situations are demand, price, price discount, rate of deterioration, shelf life, shortage, stock out, replenishment policy, etc., and the multi–echelon inventory with a centralized decision is among the most important perishable model providing that it has the information-sharing capability. Reasoning includes the adoption of a supply chain with less vertical integration.

Another inventory management policy for perishable products takes things further. Apart from the deterioration of the quality of the products, Feng [10] tried to incorporate a dynamic pricing, and replenishment policy through Pontryagin's maximum principle.

If the focus is adjusted toward the end of the supply chain of perishable products one can find a number of researches regarding the inventory policy of end or near-end parties i.e., retailers as is the case for the study conducted by Mallidis et al. [11].They tried to reduce the amount of discarded perishable products by adopting a donation policy with their replenishment policy for inventory optimization policy.

Kumar et al. [12] also optimized the warehouse management of perishable goods for retailers by generating an order proposal through Machine Learning using Random Forest Regression Algorithm by considering barriers such as supplier delivery times, and maximum and minimum number of orders. The benefits mentioned were reduction of wastages, reduction of loss, no shortages of goods, quality of food products, and enhanced sales.

According to Utama et al. [13] the Vehicle Routing Problem (VRP), which affects the distribution system's ability to discover the best routes, deserves consideration since it has the potential to boost distribution efficiency. The authors then point out one specific type of VRP for perishable products named Vehicle Routing Problem for Perishable Goods (VRPfPG). They then explain the following: the current field is divided among two objective functions namely single and multiple; the metaheuristics algorithm is the preferred solution for optimization for both functions and cost minimization if more than often was the intention.

Barma et al. [14] tried to minimize the quality degradation and delivery cost through a multi-objective VRP specifically a ring star VRP (RSVRP) for perishable products. Two types of vehicles were considered, refrigerated and general, and evolutionary algorithms NSGAII and SPEA2 were employed to solve the proposed RSVRP.

Planning of urban goods movement may be achieved through identifying, categorizing measures, and simulating the developed model as demonstrated by Russo and Comi [15]. Further, the authors have proposed a model linking retailers and final consumers and capable of determining the commodity flows in terms of quality due to restocking and service such as service time optimized route etc.

One research area of transportation planning facilities includes the transport facility location and Musolino [16] explored the decisions making process of these facilities such as Urban Distribution Centers (UDCs) considering their spatial distribution of average sales prices of different properties and transportation cost with respect to Central Business District (CBD).

Further exploring the UDC location problem, and pursuing sustainability goals Musolino et al. integrated two levels [17]. The outer level consists of a set of feasible UDC locations by considering public policies and the inner level indicates the behavior of transport behavior given a set of retailers which is influenced by restocking demand and solved through VRP simulation.

A growing research trend explores different problems regarding transportation problems by using floating car data or (FCD). Both PCP (Path Choice Problem) and VRP were used to compare the available and simulated routes all thanks to FCD by Croce et al. [18].

Croce et al. [19] again used FCD for Travel Demand Model (TDM) as a way of supporting traditional transportation models with data from new sources through the means of Information Communication System (ICTs). All these paves the way to analyze, forecast and plan the mobility patterns.

Considering the three pillars of sustainability i.e., environmental, economic and social Battaglia et al. [20] tried to integrate Travel System Models (TSM) and ICTs to estimate urban freight demand flows. FCDs are used for specification-validation-calibration of a freight zonal acquisition model.

The objective of conventional VRP is to minimize distance. It does not determine the optimal number of aggregation points needed to serve a system. Typically, VPR deals with distance and vehicle capacity constraints. The number of distribution center was stuck at only one in the cases observed. But practical solutions possess multiple distribution centers so; it is fruitless to solve VRPs without knowing the optimal number of distribution centers required for the transportation network.

Now the novelty of this research is the followings:

This research intends to improve the general VRP model. First of all, there have been several attempts to expand the reach of the basic VRP but recent investigations tend to overlook the traffic factors. Some researchers covered the area of minimizing CO_2_ emission. Traditional method of approaching a VRP model is to assume a node (AP) and try to fit the model on the system which majority of the researchers follows. In other words, they have been describing the model for existing Aggregation points. Whereas this investigation aims to integrate the aspects of optimality of aggregation points, green constraints, and traffic factors. Thus, evolving it in to the Aggregated Vehicle Routing problem or simply AVRP. Perhaps the most fascinating fact about the investigation is the amalgamation of X-means clustering algorithm to assist AVRP model in segmenting the distribution nodes. The X-means algorithm divides the nodes into optimum number of zones and assigns a service node (AP) at each centroid.

## 3 Formulation of the Aggregated Vehicle Routing Problem (AVRP) model

There may be a variety of routes open to the vehicle. The best route can save enough gas and the aggravation of being stuck in traffic. The superior path can be determined by the VRP (Vehicle Routing Problem) algorithm.

Because this investigation focuses on energy efficiency and product shelf life, we have chosen to employ a multi-objective VRP model incorporating refrigeration and non-refrigeration assumptions [14]. Other goals considered are minimal operational costs, constrained periods, stipulated capacity, and customer satisfaction.

A multi-objective VRP can be expressed with k objectives

$$Min\;f\left(x\right)=\left(f\left(x_{1}\right),f\left(x_{2}\right),\ldots \ldots .,f\left(x_{k}\right)\right)\;subject\;to\;x\;€\;X$$

Where x is a solution and X is the feasible region. Suppose, x and y are two feasible solutions and y dominates x when:

Ɐi: f_i_(x) ≤ f_i_(y) and Ǝj: f_j_(x) <f_j_(y) where i, j = 1, ….,k and k is the number of objectives. This NP problem can be solved easily using the Meta-heuristic Algorithm [13]. Here, NP means Non-Deterministic Polynomial time that is commonly used to classify the complexity of a problem.

Now, if S is a dominated solution and P is a non-dominated solution, then the MinSum and SumMin for convergence are-

MinSum = min_x €__S_,P [13]; $\sum_{i=1}^{k}fi\left(x\right)$ , where k is the number of objectives

SumMin $=\sum_{i=1}^{k}\min\;x€S,P\;fi\left(x\right)$

Range = $\sum_{i=1}^{k}\max\;{\;}_{x\;€\;S,P}\left\{f_{i}\left(x\right)\right\}\;–\;\min\;{\;}_{x\;€\;S,P}\left\{f_{i}\left(x\right)\right\}$

The range is used to calculate diversity, which is critical in multi-objective problems since it allows us to choose from many options. The vehicle's average speed, traveled distance, and time spent at the stops are predominant variables for finding the objectives [21].

### 3.1 Objective function

(1) $$\mathrm{MIN}\;Z=[\sum_{j=0}^{j=j}\sum_{i=0}^{i=i}dij\times Xij\times Fc+\sum_{j=0}^{j=j}\sum_{i=0}^{i=i}\left\{Ni\times Fr\times CF+Tij\times RL\times CE\right\}+\sum_{j=0}^{j=j}\sum_{i=0}^{i=i}\left\{Fc\times CF+\lambda ij\times CE+Iij\times Fc\times CF\right\}]$$

### 3.2 Constraints

Constraints for the aggregated VRP were constructed in four stages. In the 1st stage the basic VRP parameters were introduced. Then VRP model was specified with attribute constraints. After that limits on the energy consumption were applied in the form of green constraints. Finally, traffic factors were considered for implementing the VRP model in the heavy traffic areas.

a) Basic VRP constraints:

Vehicle Routing Problems deal with the odds related to the delivery of goods at the business or consumer level. Cost-effective distribution system often uses milk-run strategy to collect items for multiple hubs and deliver them to their specific destination. However, multiple routes may be available to reach that destination. But only one of them is optimal in most cases. These destination points are considered as nodes and the vehicle is permitted to go on a certain node at a time. The vehicle routing problem also deals with the number of vehicles that are required to obtain a expected service level. The required number of vehicles depends on the distance, cost of shipping, quantity transported, etc.

(2) $$\sum_{i,j=0}^{n}Xij=1\;;where\;i\neq j$$

(3) $$\sum_{i,j=0}^{n}Xij\leq 1\;;where\;i\neq j$$

(4) $$\sum_{i,j=0}^{n}Xij-\sum_{i,j=1}^{n}Xji=0;\;where\;i\neq j$$

(5) ∑Xn+1,d=V

(6) $$\sum_{i=0}^{n}Xi,n+2=V$$

Constraint (2) ensures that each pickup point has only one selected path, (3) indicates at most one node is permissible, aggregation of items from point 'a' to point 'b' would be equal if the vehicle travels otherwise (4). The number of vehicles is controlled by constraints (5) & (6).

b) Specific attribute of constraints:

The secondary stage of formulation of a VRP model is to set limits on the attributes related to Pick-up Points, Capacity, and Travelling Distance.

(7) Yi−Yj+(F+1)×Xij≤F

(8) $$Y^{n+2}-Y^{n+1}\leq F+1$$

(9) Ui–Uj+W×Xij+Qi≤W

(10) Un+2–Un+1=W

(11) Vi–Vj+(dij+D)×Xij≤D

(12) Vn+2–Vn−1=D

The above contraints (7) & (8) limit the number of pickup points, whereas (9) & (10) focus on capacity specification. Similarly, (11) & (12) emphasize on travelling distances.

c) Constraints of Green-VRP:

The Green VRP considers carbon emissions and tries to reduce it by minimizing the following factors.

• Vehicle driving condition
• Cold start
• Restarts

The constraints are:

(13) Fr≤T

(14) Fr≤Afc–M

(15) Nij×Xij>Fr

(16) dij≤T×Tij

(17) (CEd×D)+Tij×CEt≤E

(18) M×D<Ct

Constraints (13), (14), and (15) limit fuel consumption due to vehicle restart. (16) looks over the range of collected items per unit time from pickup nodes. But (17) equivalences electrical energy required for the refrigeration unit with the maximum available power in the vehicle. On the other hand, (18) deals with the capacity of the fuel tank and the maximum distance to travel without refueling.

d) Traffic Constraints:

The result gets fascinating if the following factors of Traffic Congestion control are synthesized with the VRP:

• Use alternative mediums
• Avoid peak hours
• Minimize marginal cost

The VRP model for perishable food primarily considers the cost of degradation. This cost estimates the loss due to product quality degradation over a given period.

Similarly, constraints associated with traffic factors are:

(19) $$Fc\leq \left\{\begin{array}{c} Rk\times \left(Afc-M\right)\;;when\;Rk=1\\ 0\;;when\;Rk=0 \end{array}\right\};Here\;Rk\;is\;a\;Binary\;variable$$

(20) $$\frac{Fc\times Cf}{Xij}+dij+\frac{\lambda ij\times CEt}{Xij}\leq Tc$$

(21) $$Iij\geq \left\{\begin{array}{c} RL\times \left(S\times D\times Tw+Sc\right)\;;when\;RL=1\\ 0\;;when\;RL=0 \end{array}\right\};\;Here\;RL\;is\;a\;Binary\;variable$$

(22) Iij≤Tt−ATt−λij

Constraint (19) manages fuel consumption due to the abrasive condition of the road. And (20) binds the components of cost. Constraints (21) & (22) set upper and lower bounds for idling time.

### 3.3 Assumptions

This model assumes all the general VRP considerations. Some additional assumptions are-

• Reverse trips will always follow the path of forward travel.
• Vehicle will only replenish from the APs.
• Fuel consumption rate is considered to be linear. In actual it may represent any function depending on the route.
• Every restart, halts, loading and unloading will always aid in increasing the internal temperature of the refrigerator.

### 3.4 Description of variables

Z = Objective function

dij = Cost of shipping a single item from i^th^ location to the following location

Xij = Total amount to be shipped

V= Number of assigned vehicles

F= Number of pick-up points to be covered

Qi = Amount collected from i^th^ location

W= Capacity of the vehicle

D = Limiting distance

Fr= Fuel required for restarts to travel from i^th^ location to the next one

T = Threshold value of fuel for restarts

Afc = Additional fuel consumption per km

M = Original Mileage when loaded

Ni= Number of restarts required to travel from i^th^ location to the next one

Tij = Loading time

CF= Unit price of fuel

CEd = Unit price of electricity as a function of distance

CEt = Unit price of electricity as a function of time

E = Energy available at the vehicle for refrigeration

Ct = Fuel capacity of the tank

Fc = Fuel consumption

λij = Idling time at i^th^ location

ATt = Average travelling time

To identify the optimal number of aggregation points, X-means clustering method is utilized. With latitude and longitude data of the service points, location and the optimal number of hubs can easily be determined.

*Location of Aggregation Points (APs):* Selecting the location for an aggregation point is not an easy task. The selection criterion is a multi-objective functionwhere the main aim is to minimize the total cost. An estimation of the location of the aggregation points can be determined using the X-means clustering approach, which potentially finds out the required number of aggregation points and their location in the globe for a given set of pickup points.

The procedure involving this technique includes determining the origins of the trips and collection hub. These origins may be any sort of marketplace where perishables are sold. And the collection hubs are the aggregation points. The main theme is to optimize the number of APs and minimize the travelling distances during milk-run process. Firstly, the locations of the origins are needed to be marked in terms of Latitude and Longitude. Then these values are converted in the 2D projection map. These 2D projection values are known as the “Northing” and “Easting” values. After that, the Northing and Easting values are to be plotted and clustered using K-means clustering with driving distances. Here the optimal value of K is unknown. So, the X-means method has proven to be a good option to do so. The number of clusters represents the optimal number of aggregation points and the centroids of such clusters embody the locations of them.

## 4 Analysis for optimal aggregation points

As the continuation of the previous section, thisarticle offers an example for the readers to help them better understand the technique. For this purpose, the authors explained the scenario in the context of Chattogram, a division in Bangladesh. The territory possesses certain characteristics suitable for fitting the overall model although the technique is suitable to implement for other regions as well.

a) Optimal Number of APs

**Fig. 1:**
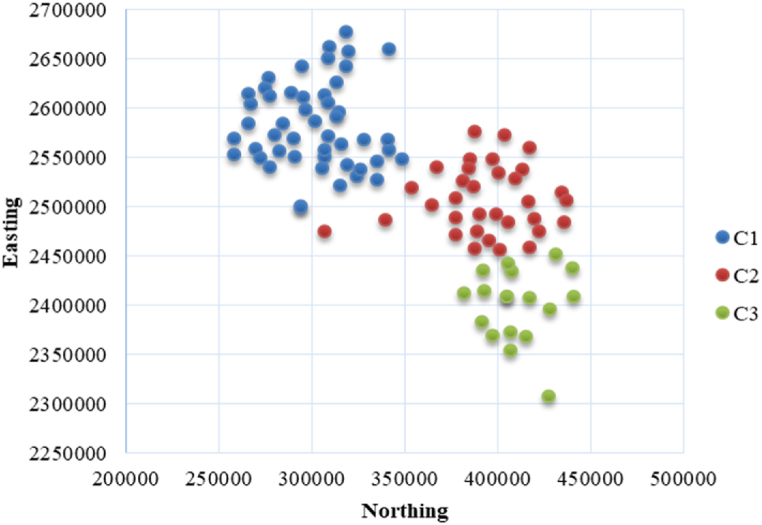
Clusters view of the Local Markets. b)Location of the APs:

Chattogram division has 11 zillas with more than 100 upazillas. Location data for all the major local markets were accumulated and converted into 2D projection values expressed in Northing and Easting. Then the 2D data were plotted in the X-Means algorithm to identify the optimal number of APs. C1, C2 and C3 legends represented in Fig. 1 illustrate relative domain of the 1st, 2nd and 3rd aggregation hubs.

Three aggregation points are required for the data set as of Fig. 1. Table 1 represents the geographic locations of the resulting cluster centroids (or aggregation points).

**Table 1:** Locations of the APs.c)Cost analysis

Symbol	Northing	Easting
AP 1	307986.20	2580682
AP 2	394270.00	2500221
AP 3	410586.60	2401048

Location of the first aggregation point (AP 1) is 307986.20, 2580682. This AP is responsible to serve the destinations indicated in Fig. 1 using blue dots. The travelling distances from AP to the cluster can be minimized using VRP approach. However, the AVRP model can be applied to minimize the cost, optimizing service time, fuel consumption and travelling distance.

### 4.1 Traditional VRP method

**Fig. 2:**
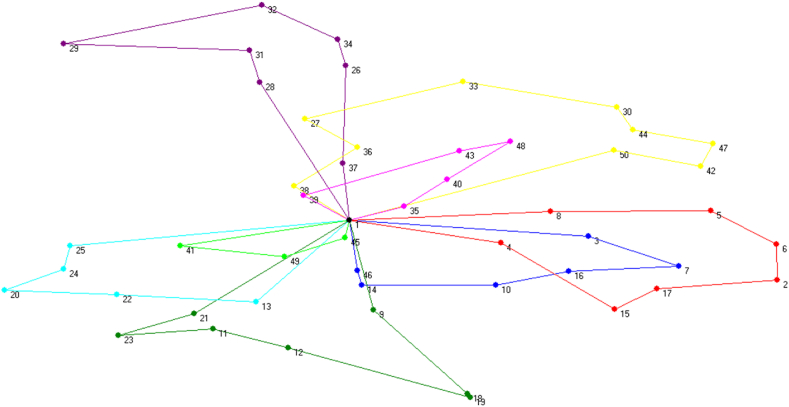
Illustration of Optimal Travelling Sequence using Traditional VRP.

As stated earlier, VRP models mainly concern in minimizing travelling distances. For the sole purpose of comparison, these distances are converted into equivalent cost. The underlying Fig. 2 shows travelling sequences of the vehicle, using closed loops of different color.

A summary is extracted from the above figure and presented in Table 2, where it is evident that the total distance traveled by the vehicle is to be 1149 Km with 8 routes or loops.

**Table 2:** Summary of output parameters in traditional VRP.

Decision Variable	Value
Travelling Distance	1149 km
Number of Trips	8
Cost Incurred	191,792 BDT

### 4.2 AVRP method

**Fig. 3:**
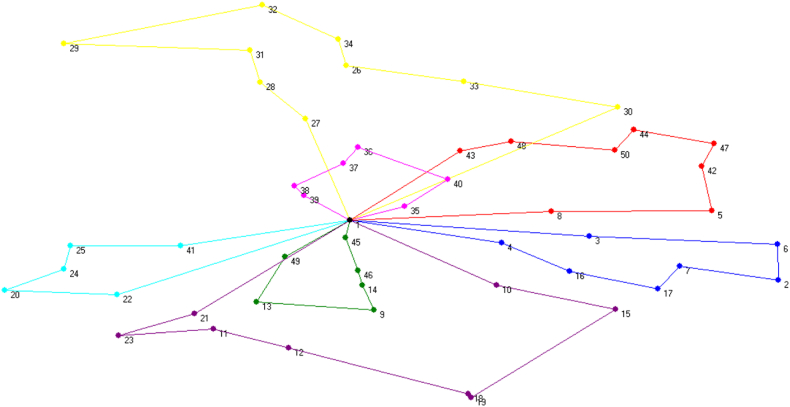
Illustration of Optimal Travelling Sequence using AVRP.

Similar calculations can be performed for AP 1, but this time with additional constraints for traffic and green factors. Here the travelling sequences have been changed to reflect these supplementary variables. Fig. 3 illustrates the rearranged routes of the AVRP model.

It is noticeable from Table 3 that, cost has been drawn down for the AVRP model as the vehicle can travel additional distances with coequal reserves.

**Table 3:** Summary of output parameters in AVRP.

Decision Variable	Value
Travelling Distance	1038 km
Number of Trips	7
Cost Incurred (Objective Function)	170,765 BDT

## 5 Discussion

An efficient transportation network design has various feasible outputs at its disposal. The concern suggests utilizing all the properties related to freight properly. This utilization is backed by the employment of the Aggregated VRP model followed by the practice of green VRP and critical traffic factors, including peak hours, route selection, and cargo type. The inclusion of aggregation points and dynamic information infrastructure allows the process to be centralized, performing multiple functions. Cluster analysis identified the number of optimal aggregation points and centroid analysis presents their apparent location. However, the technique was tested on an exemplary area. And result analysis has shown the location of three APs for Chattogram division which minimizes the driving distances. Further implementation of the AVRP has reduced travelling time, fuel consumption and distance. The AVRP model applied at AP 1 has reduced the travelling distance by 111 Km, number of trips by 1, and incurred cost by 21,000 BDT for the first aggregation point. Similarly, the process can be replicated for both AP 2 and AP 3. But locations of the APs are subject to change if new regions are added to minimize transportation cost, fuel consumption and delivery time.

## 6 Conclusion

The AVRP that has been formulated in this study addresses the concern of the modern world of sustainability and the need for a VRP that includes green characteristics in the perishable products’ supply chain. In addition, to aid the implementation, the model also considers traffic constraints that may hinder its application. The territory of Chattogram has been considered for this study and a total of three AP in three zones has been identified for the entire division through X-means clustering. Centroids of these clusters embody the geographic coordinates of the aggregation points of each class. The AVRP is applicable for each class or cluster separately. Thus, dividing the greater problem into multiple easily manageable problems. Cost calculation for delivering from AP 1 has revealed to deduct the overall cost by 10.96%. Similar cost benefits are expected from AP 2 and AP 3. Most highlighted downside of the model is its data driven. So, desired outcome of the model largely depends on precise field data. Besides the model is not suitable for existing distribution facilities as the algorithm defines the locations of the aggregation points to be the centroids of each region. In contrast, future opportunities of the AVRP depends on the criteria of model expansion, and analysis. The extension of this research can come from considering other relevant factors that may aid the implementation and sustainability. One can also develop an AVRP model for an entire region specifically for any perishable product.

## Author contribution statement

Joydev Karmakar Rahul: Conceived and designed the experiments; Performed the experiments; Wrote the paper.

Souvik Chakraborty: Performed the experiments; Analyzed and interpreted the data; Wrote the paper.

Nabila Khayer: Analyzed and interpreted the data; Contributed reagents, materials, analysis tools or data; Wrote the paper.

Md. Foysal Uddin: Conceived and designed the experiments; Contributed reagents, materials, analysis tools or data; Wrote the paper.

Maliha Rajwana Haque: Contributed reagents, materials, analysis tools or data; Wrote the paper.

## Data availability statement

Data included in article/supplementary material/referenced in article.

### Additional information

No additional information is available for this paper.

## Declaration of competing interest

The authors declare that they have no known competing financial interests or personal relationships that could have appeared to influence the work reported in this paper.
